# The IRIDICA PCR/Electrospray Ionization–Mass Spectrometry Assay on Bronchoalveolar Lavage for Bacterial Etiology in Mechanically Ventilated Patients with Suspected Pneumonia

**DOI:** 10.1371/journal.pone.0159694

**Published:** 2016-07-27

**Authors:** Kristoffer Strålin, Fredrik Ehn, Christian G. Giske, Måns Ullberg, Jonas Hedlund, Johan Petersson, Carl Spindler, Volkan Özenci

**Affiliations:** 1 Department of Infectious Diseases, Karolinska University Hospital, Stockholm, Sweden; 2 Unit of Infectious Diseases, Department of Medicine Huddinge, Karolinska Institutet, Stockholm, Sweden; 3 Department of Clinical Microbiology, Karolinska University Hospital, Stockholm, Sweden; 4 Division of Clinical Microbiology, Department of Laboratory Medicine, Karolinska Institutet, Stockholm, Sweden; 5 Unit of Infectious Diseases, Department of Medicine Solna, Karolinska Institutet, Stockholm, Sweden; 6 Department of Anesthesiology and Intensive Care, Karolinska University Hospital Solna, Stockholm, Sweden; 7 Section of Anaesthesiology and Intensive Care Medicine, Department of Physiology and Pharmacology, Karolinska Institutet, Stockholm, Sweden; Wadsworth Center, New York Department of Health, UNITED STATES

## Abstract

We studied the diagnostic performance of the IRIDICA PCR/electrospray ionization–mass spectrometry (PCR/ESI-MS) assay applied on bronchoalveolar lavage (BAL) samples, from 51 mechanically ventilated patients with suspected pneumonia, in a prospective study. In 32 patients with X-ray verified pneumonia, PCR/ESI-MS was positive in 66% and BAL culture was positive in 38% (*p* = 0.045), and either of the methods was positive in 69%. The following BAL result combinations were noted: PCR/ESI-MS+/culture+, 34%; PCR/ESI-MS+/culture-, 31%; PCR/ESI-MS-/culture+, 3.1%; PCR/ESI-MS-/culture-, 31%; kappa 0.36 (95% confidence interval (CI), 0.10–0.63). In pneumonia patients without prior antibiotic treatment, optimal agreement was noted with 88% PCR/ESI-MS+/culture+ and 12% PCR/ESI-MS-/culture- (kappa 1.0). However, in patients with prior antibiotic treatment, the test agreement was poor (kappa 0.16; 95% CI, -0.10–0.44), as 10 patients were PCR/ESI-MS+/culture-. In 8/10 patients the pathogens detected by PCR/ESI-MS could be detected by other conventional tests or PCR tests on BAL. Compared with BAL culture, PCR/ESI-MS showed specificities and negative predictive values of ≥87% for all individual pathogens, an overall sensitivity of 77% and positive predictive value (PPV) of 42%. When other conventional tests and PCR tests were added to the reference standard, the overall PPV increased to 87%. The PCR/ESI-MS semi-quantitative level tended to be higher for PCR/ESI-MS positive cases with pneumonia compared with cases without pneumonia (*p* = 0.074). In conclusion, PCR/ESI-MS applied on BAL showed a promising performance and has potential to be clinically useful in mechanically ventilated patients with suspected pneumonia. The usefulness of the method for establishment of pneumonia etiology and selection of antibiotic therapy should be further studied.

## Introduction

In severe pneumonia, selection of appropriate antibiotic therapy is essential for survival [1]. However, antibiotic selection is challenging, as pneumonia can be caused by a wide range of different pathogens [2, 3]. If the microbiological cause is detected, the patient can be given optimal, targeted antibiotic therapy. Gram stain of lower respiratory tract secretions may provide a rapid microbiological diagnosis, but this method has been associated with considerable intra- and inter-reader variability [4]. Thus, culture of lower respiratory tract secretions is currently the method of choice for detection of pneumonia etiology in most institutions [5, 6]. This method has important disadvantages, such as an analytic time of at least 24 hours, inability to detect atypical pathogens, and risk of false negativity due to antibiotic pre-treatment [5, 7]. Molecular methods are promising alternative diagnostic methods, as they may provide results within a few hours, and as they have been found to identify more microorganisms than conventional culture [8, 9].

PCR/electrospray ionization–mass spectrometry (PCR/ESI-MS) is a very interesting new molecular method, as it enables detection of DNA from >750 different bacterial species in a single test [8, 9]. The first PCR/ESI-MS system for bacterial DNA developed by Abbott, PLEX-ID, was evaluated on bronchoalveolar lavage (BAL) samples, but showed suboptimal performance [10]. Subsequently, the system was redesigned [11], and it showed improved performance when it was recently tested on lower respiratory tract secretions with a research-use instrument [12]. Compared with culture of the same samples in that study, PCR/ESI-MS showed sensitivity 84%, specificity 53%, positive predictive value (PPV) 58%, and negative predictive value (NPV) 81%. Culture was positive in 68/117 PCR/ESI-MS positive samples. For the 49 PCR/ESI-MS+/culture- cases, no additional microbiological data was provided for further evaluation [12].

In the end of 2014, Abbott´s new PCR/ESI-MS system, IRIDICA, was CE marked and became commercially available for in-vitro diagnostics in Europe. The aim of the present study was to evaluate the IRIDICA PCR/ESI-MS on BAL samples from consecutive patients with suspected pneumonia in the intensive care units (ICU) of a University Hospital. For evaluation of mismatching results, we compared the results with other conventional diagnostic tests and ran 16S PCR and species-specific PCR on the study BAL samples in selected cases. In addition, we performed analyses of cases with and without prior antibiotic therapy.

## Materials and Methods

### Patients

Samples were obtained from mechanically ventilated patients treated in the Central ICU, Thoracic ICU, Neurologic ICU, and the Extracorporeal Membrane Oxygenation Unit, of Karolinska University Hospital, Solna, Stockholm, Sweden, between 1 February and 31 May 2012. In consecutive patients who were subjected to bronchoscopy with low-volume BAL, based on the clinicians own decisions, the BAL samples were cultured and were then frozen at -80°C for future molecular diagnostics.

X-ray and/or computer tomography (CT) examinations were performed when clinically indicated. Patients were considered to have pneumonia if they had new or progressive pulmonary infiltrates on X-ray or CT, combined with clinical signs/symptoms of pneumonia, i.e. worsening of respiration and either increased volume or increased purulence of respiratory secretions. For the present study, we included pneumonia patients with BAL samples collected ± 48 h from the positive X-ray or CT examination. A patient was considered to have community-acquired pneumonia (CAP) if the clinical symptoms developed before hospital admission or within 48 h of hospital admission, and hospital-acquired pneumonia (HAP) if the clinical symptoms developed ≥48 h after hospital admission.

Patients subjected to bronchoscopy with BAL, who did not have new/progressive infiltrate combined with clinical signs of pneumonia, were considered not to have pneumonia. If repeated BAL samples were collected in those cases, only the first collected BAL sample was included in the present study.

Prior antibiotic treatment was defined as any antibiotics administered within the period 72 h–12 h prior to study bronchoscopy.

### Bronchoalveolar lavage procedure

As per routine practice, the bronchoscope was inserted through the endotracheal tube and was wedged into a bronchus, most often the bronchus of the most affected pulmonary lobe. A low-volume BAL of 10–20 mL of physiologic saline solution was injected and was subsequently aspirated.

### Microbiological analyses

All microbiological analyses were run at the Department of Clinical Microbiology, Karolinska University Laboratory, Karolinska University Hospital, Stockholm.

#### Culture of bronchoalveolar lavage

The BAL samples were routinely cultured on two regular blood agar plates, blood agar containing gentiana violet, chocolate agar, and cysteine lactose electrolyte-deficient agar. All agar plates were incubated at 37°C for 48 h. Bacterial concentration, i.e. colony-forming units (CFU)/mL was determined at 48 h. The cut-off for a positive BAL culture was 10^2^ CFU/mL.

#### Other conventional microbiological analyses

From the microbiological records, we recorded results of additional lower respiratory tract cultures, blood culture (run with a Bact/ALERT blood culturing system (BioMérieux, Durham, NC, USA)), and the *Streptococcus pneumoniae* and *Legionella pneumophila* urinary antigen tests (BinaxNOW, Alere, Waltham, USA), performed ± 5 days from the study bronchoscopy.

#### IRIDICA PCR/electrospray ionization–mass spectrometry

PCR/ESI-MS was performed from November 2014 to February 2015, with an IRIDICA BAC LRT system (Part Number: 08N40-010, Ibis Biosciences, Abbott, Des Plaines, USA), according to the manufacturer's instructions. This assay was designed to identify DNA from >750 different bacterial species, including the antibiotic resistance markers *mecA*, *vanA*, *vanB*, and *kpc*. Briefly, 0.1 mL of BAL fluid was diluted with Tris-EDTA buffered solution to a sample volume of 5 mL, and the sample was lysed using the IRIDICA bead-beater. DNA was extracted with the IRIDICA DNA Prep Kit, using the automated extraction system. Purified DNA in buffer was automatically distributed by the IRIDICA sample prep into the 16-well IRIDICA BAC LRT Assay Strips containing PCR reagents and primers for 16 PCR reactions. PCR was performed on the IRIDICA Thermal Cycler using a preloaded PCR amplification protocol. After PCR amplification, the IRIDICA BAC LRT Assay Stips were loaded onto the IRIDICA desalter, which purified DNA to remove substances which may interfere with mass spectrometry. Following desalting, plates were loaded onto the IRIDICA mass spectrometer. Purified amplicons were injected one well at a time into an electrospray ionization time-of-flight mass spectrometer for determination of the molecular mass of the amplicons. The resulting information was used for species identification by automated database comparison. The IRIDICA system had an analytic time from original sample to result of 6 h.

#### Semi-quantitative level determined by PCR/electrospray ionization–mass spectrometry

Each well of the IRIDICA BAC LRT Assay Strip contains an internal control template at a known concentration. This template generates an amplicon with a known signature. Comparison of the relative concentrations of amplicons from this template and from detected organisms is reported as a “level”, which represents a semi-quantitative marker of the DNA content of the sample. For each positive PCR/ESI-MS result in the study, such a level was generated.

#### 16S PCR and species-specific PCR

After analysis with PCR/ESI-MS, BAL samples positive by PCR/ESI-MS or culture without support from other conventional diagnostic tests were subjected to direct 16S rRNA gene sequencing, with special focus on polybacterial samples and interpretation of mixed DNA chromatograms [13]. In patients with positive PCR/ESI-MS findings of *S*. *pneumoniae*, *Staphylococcus aureus*, and/or *Haemophilus influenzae*, with no support from conventional diagnostic tests, species-specific PCR [14, 15] was run.

#### Normal respiratory flora by culture, PCR/electrospray ionization–mass spectrometry, and 16S PCR

In culture, PCR/ESI-MS, and 16S PCR of BAL, the following species were considered to belong to the normal respiratory flora; alpha-hemolytic streptococci, coagulase-negative staphylococci, *Enterococcus* spp., *Neisseria* spp., *Corynebacterium* spp., and *Candida* spp.

Normal respiratory flora was considered as negative in the analyses of the study.

### Data and statistical analysis

Correlation and significance of relationships between the studied parameters was assessed using Chi-square test, Fisher´s exact test, and Mann-Whitney U test. *p*-values below 0.05 were considered statistically significant. For test agreement, a κ value with a 95% confidence interval (CI) was determined.

### Ethical considerations

The Regional Ethics Committee in Stockholm approved the study and approved inclusion of patients in the study without written informed consent, as no intervention or additional procedures or samplings were performed (approval number, 2011/1774-31/2).

## Results

### Patients

**Fig 1:**
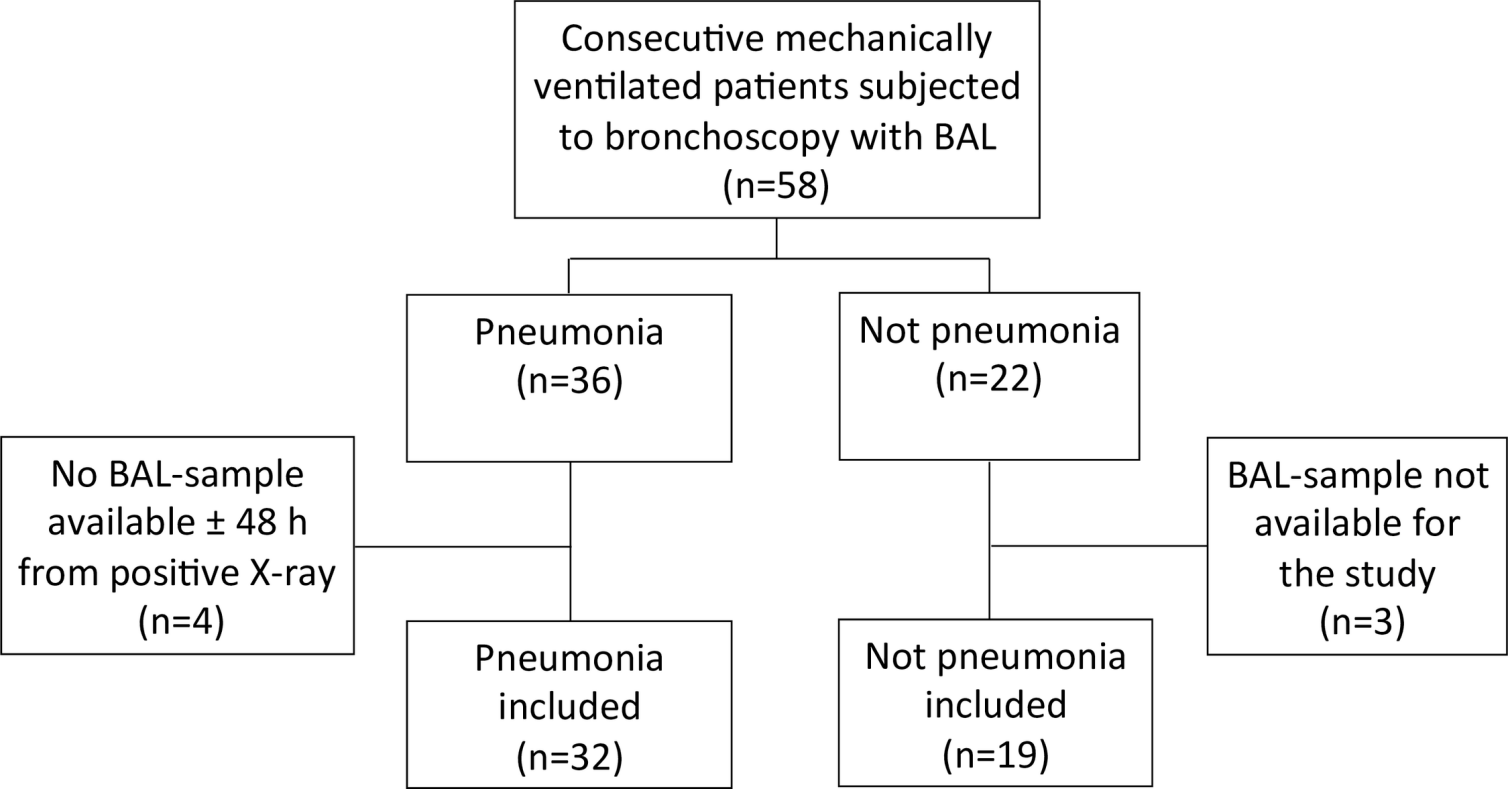
Flow shart of the study patients. ICU, intensive care unit; BAL, bronchoalveolar lavage.

Fifty-eight mechanically ventilated patients were subjected to bronchoscopy and 51 patients were included (Fig 1), including 32 patients with X-ray verified pneumonia (15 patients with CAP and 17 patients with HAP), and 19 patients without pneumonia. Table 1 shows patient characteristics. All CAP patients and 9 HAP patients (53%) were treated with antibiotics prior to study bronchoscopy.

**Table 1:** Characteristics of included patients. All patients were mechanically ventilated.

Characteristics	Total (*n* = 51)	Pneumonia (*n* = 32)	Not pneumonia (*n* = 19)
Female	24 (47)	16 (50)	8 (42)
Median age (IQR)	50 (16–68)	49 (4–69)	53 (44–68)
Comorbidity			
- Congestive heart disease	6 (12)	2 (6)	4 (21)
- Chronic lung disease	9 (18)	5 (16)	4 (21)
- Neoplastic disease	7 (14)	6 (19)	1 (5)
- Liver disease	3 (6)	1 (3)	2 (11)
- Cerebrovascular disease	1 (2)	0	1 (5)
- Renal disease	0	0	0
- Immunosuppressive disease	5 (10)	4 (12)	1 (5)
Antibiotic therapy within 72 h prior to sampling	40 (78)	24 (75)	16 (84)
Median days in hospital prior to bronchoscopy (IQR)	7 (3–14)	7 (2–15)	6 (3–14)
Median days in intensive care unit prior to bronchoscopy (IQR)	4 (1–7)	3 (1–7)	5 (2–8)
Median days of mechanical ventilation prior to bronchoscopy (IQR)	5 (0–8)	4 (0–8)	5.5 (0–10)
< 48 h duration of mechanical ventilation prior to sampling	23 (45)	8 (25)	15 (79)
≥ 48 h duration of mechanical ventilation prior to sampling	28 (55)	24 (75)	4 (21)

Data are presented as number (%), unless otherwise indicated; IQR, inter-quartile range.

### BAL culture and PCR/electrospray ionization–mass spectrometry in pneumonia patients

Among 32 pneumonia patients, either BAL culture or PCR/ESI-MS was positive in 22 patients (69%), as BAL culture was positive in 12 patients (38%) and PCR/ESI-MS was positive in 21 patients (66%; *p =* 0.045). The result combinations of PCR/ESI-MS and BAL culture are shown in Table 2. PCR/ESI-MS+/culture- results were noted in 7/15 CAP cases (47%) and in 3/17 HAP cases (18%).

**Table 2:** Positive and negative results of IRIDICA PCR/electrospray ionization–mass spectrometry (PCR/ESI-MS) and culture of bronchoalveolar lavage in mechanically ventilated patients with pneumonia.

Result combination	All pneumonia patients (n = 32), *n* (%)	Patients *without* prior antibiotics (*n* = 8), *n* (%)	Patients *with* prior antibiotics (*n* = 24), *n* (%)
PCR/ESI-MS+, culture+	11 (34)	7 (88)	4 (17)
PCR/ESI-MS+, culture-	10 (31)	0	10 (42)
PCR/ESI-MS-, culture+	1 (3.1)	0	1 (4.2)
PCR/ESI-MS-, culture-	10 (31)	1 (12)	9 (38)
κ for test agreement ^a^ (95% confidence interval)	0.36 (0.10–0.63)	1.0 (1.0–1.0)	0.16 (-0.11–0.44)

^a^ Agreement regarding positive and negative results, not regarding species-specific results.

Table 2 also shows the result combinations of patients with and without prior antibiotic treatment. The test agreement between PCR/ESI-MS and culture was high in patients without, and poor in patients with prior antibiotic treatment. Among cases without and with prior antibiotic treatment, BAL culture was positive in 88% (7/8) and 21% (5/24; *p* = 0.0016) and PCR/ESI-MS was positive in 88% (7/8) and 58% (14/24; *p* = 0.21), respectively. All 10 cases with PCR/ESI-MS+/culture- results had received prior antibiotic treatment.

Tables 3 and 4 show individual results of CAP and HAP patients. In 8/10 patients with PCR/ESI-MS+/culture- results (6 CAP and 2 HAP cases), the detected pathogens were also detected by conventional tests or PCR tests.

**Table 3:** Individual mechanically ventilated patients with *community-acquired pneumonia* with positive result obtained with either IRIDICA PCR/electrospray ionization–mass spectrometry (PCR/ESI-MS) or culture on bronchoalveolar lavage (BAL). Support for positive PCR/ESI-MS results is indicated in bold.

Sex, Age in years	Prior anti-biotic therapy	Study BAL sample				Other conventional tests on samples collected ± 5 d from the study BAL sample	
		PCR/ESI-MS (semi-quantitative level)	BALCulture (CFU/mL)	16S PCR	Species-specific PCR	Routine cultures	Urinary antigen test
m, 0	Y	*Klebsiella pneumoniae* (144)	***K*. *pneumoniae*** (>10^4^)	ND	ND	***K*. *pneumoniae*** in blood culture and LRT culture	ND
f, 35	Y	*Enterobacter cloacae* (20)	***E*. *cloacae*** (10^2^−10^3^)	ND	ND	ND	*Streptococcus pneumoniae-Legionella pneumophila-*
m, 1	Y	*Stenotrophomonas maltophilia* (146)	***S*. *maltophilia*** (10^2^−10^3^)	ND	ND	negative	ND
m, 44	Y	*Streptococcus pyogenes* (177), *Staphylococcus aureus* (6)	***S*. *pyogenes*** (10^2^−10^3^)	ND	***S*. *aureus****+*	***S*. *pyogenes***in blood culture and LRT culture	*S*. *pneumoniae-L*. *pneumophila-*
f, 16	Y	*S*. *pneumoniae* (322)	negative	ND	ND	negative	***S*. *pneumoniae****+L*. *pneumophila-*
f, 60	Y	*S*. *pneumoniae* (359)	negative	ND	ND	negative	***S*. *pneumoniae+****L*. *pneumophila-*
f, 60	Y	*L*. *pneumophila* (208)	negative	ND	ND	negative	*S*. *pneumoniae-****L*. *pneumophila+***
m, 48	Y	*S*. *pneumoniae* (162), *Tropheryma whipplei* (283)	negative	***T*. *whipplei***	***S*. *pneumoniae*+**	negative	*S*. *pneumoniae-L*. *pneumophila-*
m, 62	Y	*S*. *aureus* (27)	negative	ND	***S*. *aureus+***	negative	ND
m, 9	Y	*S*. *pyogenes* (194)	negative	***S*. *pyogenes***	ND	negative	*S*. *pneumoniae-L*. *pneumophila-*
m, 0	Y	*Acinetobacter baumannii* (24)	negative	negative	ND	negative	*S*. *pneumoniae-L*. *pneumophila-*

CFU, colony-forming units; Y, yes; ND, not done; negative, no detected pathogens or normal flora; LRT, lower respiratory tract.

**Table 4:** Individual mechanically ventilated patients with *hospital-acquired pneumonia* with positive results obtained with either IRIDICA PCR/electrospray ionization–mass spectrometry (PCR/ESI-MS) or culture on bronchoalveolar lavage (BAL). Support for positive PCR/ESI-MS results is indicated in bold.

Sex, Age in years	Prior antibiotic therapy	Study BAL sample		Other lower respiratory tract cultures ± 5 d from the study BAL sample
		PCR/ESI-MS (semi-quantitative level)	BALCulture (CFU/mL)	
f, 50	N	*Staphylococcus aureus* (140)	***S*. *aureus*** (>10^4^)	***S*. *aureus***
f, 79	N	*S*. *aureus* (202)	***S*. *aureus*** (>10^4^)	negative
m, 69	N	*Pseudomonas aeruginosa* (124)	***P*. *aeruginosa*** (>10^4^)	***P*. *aeruginosa***
f, 46	N	*Enterobacter cloacae* (344)	***E*. *cloacae*** (>10^4^)	***E*. *cloacae***
m, 68	N	*Serratia marcescens* (5)	***S*. *marcescens*** (10^3^−10^4^)	***S*. *marcescens***, *Haemophilus influenzae*
f, 31	N	*H*. *influenzae* (138)	***H*. *influenzae*** (>10^4^), *E*. *cloacae* (10^3^−10^4^)	*E*. *cloacae*
m, 74	N	*H*. *influenzae* (150)	*E*. *cloacae* (>10^4^)	***H*. *influenzae***, *E*. *cloacae*
f, 71	Y	*P*. *aeruginosa* (360)	negative	***P*. *aeruginosa***
f, 68 ^a^	Y	*S*. *aureus* (22), *Streptococcus pneumoniae/Streptococcus agalactiae* (37)	negative	***S*. *aureus***
m, 72 ^b^	Y	*S*. *pyogenes* (260)	negative	negative
m, 50 ^b^	Y	negative	*E*. *cloacae* (10^3^−10^4^)	negative

CFU, colony-forming units; N, no; Y, yes; negative, no detected pathogens or normal flora.

^a^ 16S PCR and species-specific PCR for *S*. *pneumoniae* on BAL, and *S*. *pneumoniae* urinary antigen test were negative.

^b^ 16S PCR on BAL was negative.

Compared with BAL culture, PCR/ESI-MS showed specificities of ≥87% and negative predictive values of ≥90% for all individual pathogens. The overall sensitivity was acceptable, 77% (10/13), but it should be noted that false-negative PCR/ESI-MS results were noted only for 3 cases of *Enterobacter cloacae*. The overall PPV was low, 42% (10/24). However, with an expanded reference standard, it increased. The PPV increased to 67% (16/24) when results of conventional diagnostic tests were added to the reference standard, and to 87% (21/24) when results of PCR tests were also added.

Table 5 shows combined results of culture and PCR/ESI-MS on BAL for individual pathogens.

**Table 5:** Combined results of IRIDICA PCR/electrospray ioization-mass spectrometry (PCR/ESI-MS) and culture on bronchoalveolar lavage (BAL) for individual pathogens (with > 1 positive result), in mechanically ventilated patients with and without pneumonia.

Bacterial pathogen	Pneumonia			Not pneumonia		
	PCR/ESI-MS+ Culture+	PCR/ESI-MS+ Culture-	PCR/ESI-MS- Culture+	PCR/ESI-MS+ Culture+	PCR/ESI-MS+ Culture-	PCR/ESI-MS- Culture+
*Staphylococcus aureus*	2	3	0	0	3	0
*Streptococcus pneumoniae*	0	4^a^	0	0	1	0
*Streptococcus pyogenes*	1	2	0	0	0	0
*Haemophilus influenzae*	1	1	0	1	2	0
*Enterobacter cloacae*	2	0	3	2	1	0
*Klebsiella pneumoniae*	1	0	0	0	1	0
*Pseudomonas aeruginosa*	1	1	0	1	0	1

^a^ One patient was PCR/ESI-MS positive for *S*. *pneumoniae/Streptococcus agalactiae* and was presented as a *S*. *pneumoniae* case in this table.

### Culture and PCR/electrospray ionization–mass spectrometry in patients without pneumonia

In 19 patients without pneumonia, BAL culture was positive in 5 patients (26%) and PCR/ESI-MS was positive in 10 patients (53%; *p* = 0.18), and either of the methods was positive in 10 patients (53%). The following combined test results were noted: PCR/ESI-MS+/culture+, n = 5 (25%); PCR/ESI-MS+/culture-, n = 5 (25%); PCR/ESI-MS-/culture+, n = 0; PCR/ESI-MS-/culture-, n = 9 (47%); κ for test agreement, 0.49 (95% CI, 0.15–0.82).

Individual microbiological results of patients without pneumonia are shown in Table 6. Among 5 patients with PCR/ESI-MS+/culture- results, the detected pathogen was supported by positive PCR tests in 2 cases. Table 5 shows combined results of PCR/ESI-MS and culture for different species.

**Table 6:** Individual mechanically ventilated patients without pneumonia with positive result obtained with either IRIDICA PCR/electrospray ionization–mass spectrometry (PCR/ESI-MS) or culture on bronchoalveolar lavage (BAL). Support for positive PCR/ESI-MS results is indicated in bold.

Sex, Age in years	Prior antibiotic therapy	Study BAL sample				Other conventional tests on samples collected ± 5 d from the study BAL sample
		PCR/ESI-MS (quantitative level)	BALCulture (CFU/mL)	16S PCR	Species-specific PCR	
m, 81	N	*Pseudomonas aeruginosa* (207)	***P*. *aeruginosa*** (>10^4^)	ND	ND	***P*. *aeruginosa*** in LRT culture
f, 62	N	*Haemophilus influenzae* (136)	negative	negative	***H*. *influenzae*+**	negative
m, 45	N	*H*. *influenzae* (16)	negative	*Haemophilus parahaemolyticus*	*H*. *influenzae****-***	*E*. *cloacae*in LRT culture
f, 53	Y	*Enterobacter cloacae* (256)	***E*. *cloacae*** (>10^4^)	ND	ND	negative
f, 72	Y	*E*. *cloacae* (185)	***E*. *cloacae*** (10^3^−10^4^)	ND	ND	negative
m, 46	Y	*H*. *influenzae* (80), *Staphylococcus aureus* (52)	***H*. *influenza***(>10^4^)	ND	***S*. *aureus*+**	negative
m, 74	Y	*Klebsiella pneumoniae* (173), *S*. *aureus* (5)	negative	***K*. *pneumoniae***	***S*. *aureus*+**	negative
f, 75	Y	*E*. *cloacae* (3)	*P*. *aeruginosa* (10^3^−10^4^)	***E*. *cloacae***	ND	negative
m, 44	Y	*S*. *aureus* (1)	negative	negative	*S*. *aureus***-**	*P*. *aeruginosa* in LRT culture
f, 47	Y	*Streptococcus pneumoniae* (18)	negative	negative	*S*. *pneumoniae***-**	negative ^a^

CFU, colony-forming units; N, no; Y, yes; ND, not done; negative, no detected pathogens or normal flora.

^a^ *S*. *pneumoniae* urinary antigen test not performed.

### Semi-quantitative level of PCR/electrospray ionization–mass spectrometry

**Fig 2:**
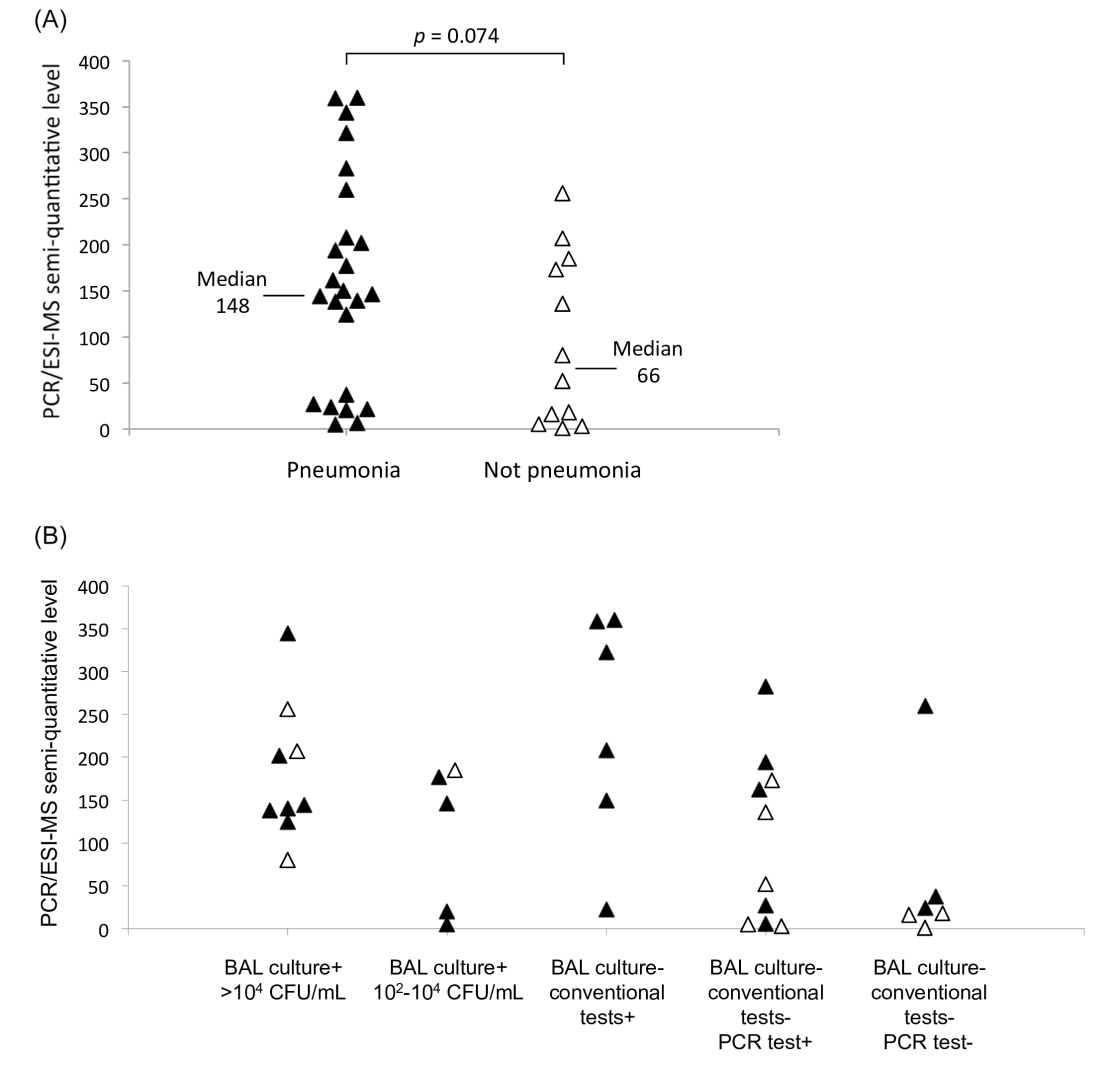
Semi-quantitative levels of IRIDICA PCR/electrospray ionization-mass spectrometry (PCR/ESI-MS) on bronchoalveolar lavage (BAL). (A) Results of cases with and without pneumonia; (B) Results related to other microbiological results. Black triangles, pneumonia; white triangles, not pneumonia. CFU, colony-forming units.

Semi-quantitative levels of PCR/ESI-MS are shown in Tables 3–4 and 6. The levels tended to be higher for 24 positive results of pneumonia patients than for 12 positive results of patients without pneumonia, *p* = 0.074 (Fig 2A). However, no correlation with semi-quantitative culture results or other microbiological results could be noted (Fig 2B).

### Resistance genes detected by PCR/electrospray ionization–mass spectrometry

PCR/ESI-MS was positive for *mecA* in 19 study samples, including 5 samples PCR/ESI-MS positive for *S*. *aureus* and 14 samples with PCR/ESI-MS showing normal respiratory flora. Among 5 samples PCR/ESI-MS positive for *S*. *aureus* and *mecA*, BAL culture was positive for methicillin-sensitive *S*. *aureus* in 2 cases. A third patient was positive for methicillin-sensitive *S*. *aureus* in another lower respiratory tract culture. No methicillin-resistant *S*. *aureus* was isolated from any study patient.

No study sample was PCR/ESI-MS positive for *vanA*, *vanB*, or *kpc*, or culture positive for vancomycin resistant enterococci or carbapenemase secreting Gramnegatives.

## Discussion

In this study of mechanically ventilated patients with suspected pneumonia, PCR/ESI-MS on BAL showed a promising performance for detection of bacterial etiology.

BAL is a suitable sample type for detection of microbiological cause in pneumonia, as BAL fluid covers respiratory epithelium at or close to the site of the infection. BAL culture has been found to reliably identify qualitatively and quantitatively microorganisms present in lung segments [16]. However, a problem in clinical practice is that antibiotics taken prior to sampling may produce false negative culture results. When Prats et al. [17] performed repeated bronchoscopy on culture positive patients with ventilator-associated pneumonia, the culture positivity rate decreased to 54% after 24 h and to 20% after 48 h of antibiotic treatment. Accordingly, the present study showed that BAL culture was significantly less often positive in patients with than in patients without prior antibiotic therapy.

Our group [18] previously found that PCR applied on BAL was significantly more often positive for *S*. *pneumoniae* than BAL culture (31% vs. 2.9%), in patients with prior antibiotic treatment. Baudel et al. [19] found that the multiplex PCR test LightCycler SeptiFast (Roche Diagnostics) on BAL was significantly more often positive than BAL culture in both antibiotic treated and un-treated patients. PCR was positive in 66% of their overall study and in 64% of antibiotic pre-treated patients. In the study by Vincent et al. [12], 75% of the patients were treated with antibiotics at enrolment, and PCR/ESI-MS was more often positive than culture on lower respiratory tract secretions (63% versus 44%).

Accordingly, the present study showed that PCR/ESI-MS was positive for bacterial pathogens more often than culture. When PCR/ESI-MS was added to culture, the frequency of pneumonia patients with a bacterial finding in BAL increased from 38% to 69%. As the positivity rate of culture and PCR/ESI-MS was identical in patients without prior antibiotic treatment, the improved positivity rate resulted exclusively from patients with prior antibiotic therapy (Table 2). The fact that the detected pathogen was identified by other diagnostic methods in 8/10 PCR/ESI-MS+/culture- pneumonia cases indicates that PCR/ESI-MS detected a pathogen that was or had recently been present in the lower respiratory tract. However, the study was not designed to evaluate if the detected pathogen represented the pathogen that caused the patient´s infection or if it was just a colonizer.

Colonization with respiratory pathogens is common in mechanically ventilated patients. Berdal et al. [20] collected repeated BAL samples from mechanically ventilated ICU patients who had any diagnosis other than pneumonia, and found pneumonia pathogens in BAL samples at ≥ 10^4^ CFU/mL in 57% of the patients, including 36% of patients who did not develop X-ray infiltrates. Accordingly, many patients without pneumonia in the present study had positive culture and PCR/ESI-MS on BAL (Table 6).

In order to differentiate between infection and colonization, a cut-off limit of 10^4^ CFU/mL has been proposed for BAL culture [16]. However, as rapid decreases of bacterial counts have been found during antibiotic treatment [17], it has been suggested that the cut-off limit should be decreased in antibiotic treated patients [21]. In addition, a recent meta-analysis did not find evidence that the use of quantitative cultures of respiratory secretions resulted in reduced mortality, reduced time in ICU and on mechanical ventilation, or higher rates of antibiotic changes, when compared to qualitative cultures in patients with ventilator-associated pneumonia [22]. When we studied semi-quantitative data of PCR/ESI-MS on BAL in the present study, there was only a tendency that pneumonia patients had higher semi-quantitative levels than cases without pneumonia (Fig 2A). No correlation with other microbiological results was noted (Fig 2B). Thus, the usefulness of semi-quantitative data of PCR/ESI-MS on BAL appears to be limited.

An interesting finding from this study was that PCR/ESI-MS was positive for *S*. *aureus* and *mecA* in 2 samples that were culture positive for methicillin-sensitive *S*. *aureus*. The most probable explanation is that the *mecA* genes were harboured by coagulase-negative staphylococci (i.e. normal flora) in the samples. As it is difficult to link resistance markers to detected bacterial pathogens by currently available molecular methods, combined detection of *S*. *aureus* and *mecA* in BAL samples by PCR/ESI-MS should be interpreted with caution.

An advantage with the PCR/ESI-MS technique compared with multiplex PCR with species-specific primers is that it can detect a large number of different pathogens, including unexpected pathogens. In this study, *L*. *pneumophila* and *Tropheryma whipplei* were detected by PCR/ESI-MS. Both pathogens were also detected by other diagnostic methods (Table 3). *T*. *whipplei* is an uncommon cause of pneumonia [23].

The major limitation of this study was its small sample size. Thus, we could not evaluate the performance of PCR/ESI-MS for detection of etiology in CAP or HAP, or for detection of individual pathogens.

We conclude that PCR/ESI-MS showed a promising performance and has potential to be clinically useful in mechanically ventilated patients with suspected pneumonia. The usefulness of the method for establishment of pneumonia etiology and selection of antibiotic therapy should be further studied.
